# Supplementary material for: Discrimination between Complete versus Non-Complete Pathologic Response to Neoadjuvant Therapy Using Ultrasensitive Mutation Analysis: A Proof-of-Concept Study in BRCA1-Driven Breast Cancer Patients
Source: Int J Mol Sci. 2023 Jan 18;24(3):1870. doi: 10.3390/ijms24031870 (PMC9914941; doi:10.3390/ijms24031870)
Supplement: Supplementary file 1 [file ijms-24-01870-s001.zip › ijms-2137228-supplementary.pdf]

Supplementary Table S1. Clinical description of the NACT patients

Clinical characteristics	Non-carriers (n = 204)	*BRCA1* carriers (n = 25)	Total (n = 229)
**Mean age (range)**	55.6 (26-89)	49.2 (28-76)	54.9 (26-89)
**Clinical tumor stage**			
T1-2	103 (50.5%)	20 (80.0%)	123 (53.7%)
T3-4	101 (49.5%)	5 (20.0%)	106 (46.3%)
**Clinical nodal stage**			
N0	32 (15.7%)	7 (28.0%)	39 (17.0%)
N1-3	172 (84.3%)	18 (72.0%)	190 (83.0%)
**Histology**			
Invasive ductal carcinoma	202 (99.0%)	25 (100%)	227 (99.1%)
Medullary carcinoma	2 (1.0%)	0 (0)	2 (0.9%)
**Subtype**			
Luminal A	40 (19.6%)	0 (0)	40 (17.5%)
Luminal B	90 (44.1%)	4 (16.0%)	94 (41.0%)
HER2	35 (17.2%)	0 (0)	35 (15.3%)
Triple-negative	39 (19.1%)	21 (84.0%)	60 (26.2%)
**Number of NACT cycles**			
Median	6	4	6
Range	2-16	2-8	2-16
**Chemotherapy type**			
Platinum-containing schemes	19 (9.3%)	18 (72.0%)	37 (16.2%)
Taxanes + platinum	15 (7.4%)	9 (36.0%)	24 (10.5%)
Platinum monotherapy	1 (0.5%)	8 (32.0%)	9 (3.9%)
Taxanes + platinum + anthracyclines	3 (1.5%)	1 (4.0%)	4 (1.7%)
Non-platinum regimens	185 (90.7%)	7 (28.0%)	192 (83.8%)
Taxanes + anthracyclines	79 (38.7%)	3 (12.0%)	82 (35.8%)
Anthracyclines without taxanes	69 (33.8%)	4 (16.0%)	73 (31.9%)
Taxane monotherapy	12 (5.9%)	0 (0)	12 (5.2%)
Other	25 (12.3%)	0 (0)	25 (10.9%)
**RECIST 1.1**	**(n = 189)**	**(n = 19)**	**(n = 208)**
CR	4 (2.1%)	2 (10.5%)	6 (2.9%)
PR	113 (59.8%)	14 (73.7%)	127 (61.0%)
SD	67 (35.4%)	3 (15.8%)	70 (33.6%)
PD	5 (2.6%)	0 (0)	5 (2.4%)
**Pathologic complete response (pCR)**	**(n = 192)**	**(n = 24)**	**(n = 216)**
Yes	29 (15.1%)	13 (54.2%)	42 (19.4%)
No	163 (84.9%)	11 (45.8%)	174 (80.6%)
**Miller-Payne score**	**(n = 141)**	**(n = 21)**	**(n = 162)**
1	22 (16.2%)	1 (4.8%)	23 (14.2%)
2	47 (32.3%)	4 (19.0%)	51 (31.5%)
3	28 (20.8%)	4 (19.0%)	32 (19.8%)
4	22 (14.6%)	1 (4.8%)	23 (14.2%)
5	22 (16.2%)	11 (52.4%)	33 (20.4%)

Supplementary Table S2. Primers and probes used for *TP53* ddPCR

Mutation	Sequence 5’→3’
**R213X [c.637C>T]**	
F	GGAAATTTGCGTGTGGAGTAT
R	CAGGCGGCTCATAGGGCA
P wt	[R6G]CACTATGTCGAAAAGTGTTTCTG[BHQ1]
P mut	[FAM]CACTATGTCAAAAAGTGTTTCTG[BHQ1]
**I255N [c.764T>A]**	
F	ACATGTGTAACAGTTCCTGC
R	CAAGTGGCTCCTGACCTG
P wt	[R6G]TCCTCACCATCATCACACTG[BHQ1]
P mut	[FAM]TCCTCACCATCAACACACTG[BHQ1]
**Y234C [c.701A>G]**	
F	CTAGGTTGGCTCTGACTGTA
R	GGATGGGCCTCCGGTTCA
P wt	[R6G]CCACCATCCACTACAACTACAT[BHQ1]
P mut	[FAM]CCACCATCCACTGCAACTACAT[BHQ1]
**M237I [c.711G>A]**	
F	CTAGGTTGGCTCTGACTGTA
R	TGGCAAGTGGCTCCTGAC
P wt	[FAM]ACTACATGTGTAACAGTTCCTGC[BHQ1]
P mut	[R6G]ACTACATATGTAACAGTTCCTGC[BHQ1]
**Y220C [c.659A>G]**	
F	TGGAGAGACGACAGGGCT
R	TCCCAGAGACCCCAGTTG
P wt	[FAM]TGGTGCCCTATGAGCCGCCTGAG[BHQ1]
P mut	[R6G]TGGTGCCCTGTGAGCCGCCTGAG[BHQ1]
**R273L [c.818G>T]**	
F	TAATCTACTGGGACGGAACA
R	TCCTCTGTGCGCCGGTCT
P wt	[FAM]CACAAACACGCACCTCAAAGC[BHQ1]
P mut	[R6G]CACAAACAAGCACCTCAAAGC[BHQ1]
**R273H [c.818G>A]**	
F	TAATCTACTGGGACGGAACA
R	TCCTCTGTGCGCCGGTCT
P wt	[FAM]CACAAACACGCACCTCAAAGC[BHQ1]
P mut	[R6G]CACAAACATGCACCTCAAAGC[BHQ1]
**S149fs*21 [c.445delT]**	
F	CAAGACCTGCCCTGTGC
R	TGCTGTGACTGCTTGTAGA
P wt	[R6G]T+GTGG+AA+TC+AA+CC[BHQ1]*
P mut	[FAM]T+G+TGG+ATC+AA+CC[BHQ1]*

*Probes with LNA bases
